# Gunshot residues found at the exit wound: a case report

**DOI:** 10.1007/s00414-022-02842-w

**Published:** 2022-06-03

**Authors:** Anja Weber, Beat P. Kneubuehl, Walter Rabl

**Affiliations:** 1 Institute of Legal Medicine, Medical University of Innsbruck, Müllerstraße 44, 6020 Innsbruck, Austria; 2 Forensic Physics and Ballistics, Institute of Forensic Medicine, University of Bern, Bern, Switzerland; 3 Bpk Consultancy GmbH, Thun, Switzerland

**Keywords:** Gunshot wound, Gunshot residues, Exit wound, Ballistics, Suicide

## Abstract

Gunshot residues (GSRs) play an important role in forensic investigations of gun-related violence. The presence of GSRs has been described to help to identify the bullet entry area, as it was supposed not to be found at exit wounds. This report details the suicidal headshot of an 84-year-old male where unburned tube-like, cuboid and flake-formed powder particles have been found not only at the inside of the muzzle but also circular around the exit wound. With very short-barrelled weapons, it must be expected that part of the propellant charge leaves the barrel unburned behind the bullet. In contrast to that, the barrel length of the used weapon should lead to a complete burn-up of powder particles. The surprisingly large number of unburned powder particles present at the exit wound of the injury gave reason for further investigation to understand the underlying ballistic aspects and outlines the importance of having a close look at incidence scene photos during an investigation.

## Introduction

By firing a gun, the bullet passes through the barrel followed by flame, gases, metallic particles, soot and to some extent (partially) burned gunpowder. These gunshot residues (GSRs) play an important role in forensic investigations of gun-related violence, as they may help to analyse who has been involved in the shooting [1–5], to differentiate entrance and exit wounds, to determine muzzle-to-target distance and muzzle-to-target angle [6]. The chemical composition of GSRs has even been recognized as a kind of “signature” for the fired ammunition and its manufacturer [4]. Furthermore, GSRs are also detectable on the hands after mere handling and/or loading of a gun without shooting and can even be transmitted from one person to another, for example from police officers to suspects [1, 7, 8].

For any crime scene investigation, it is crucial to preserve the scene as much as possible and not to disturb any physical evidence — especially in case of trace evidence, like GSRs, where minimal impact can destroy essential evidence. Scene photographs should document the original position of the body and any evidence. GSRs should subsequently be secured by forensic experts and undergoing further chemical tests [9]. The different ballistic aspects are worked up by different scientific disciplines, whereas internal and external ballistics are covered by specialists from the police or scientific fields like physics or mathematics. In contrast to that, end/wound ballistics is predominantly the task area of legal medicine. Main part thereby is the examination of the body, whereas the number of gunshot wounds, the shooting distance and the bullet entry angle are determined. For the determination of the shooting distance and discrimination of entry and exit wound, GSRs play an important role [10]. In the vast majority of close-range shots, GSRs are found predominantly around the entrance wound or, in cases of a contact shot, they are also located subcutaneously at the entrance wound margins. In some cases, depending on muzzle-to-skin distance and type of gun, gunpowder is even “tattooing” the skin, which is unique for bullet entry regions and not found at exit wounds [11–13]. GSRs have been extensively described to help to discriminate entrance and exit wounds [13–15]. The presence of GSRs has even been postulated to be an exclusion criterion for exit wounds [16, 17]. Nowadays, there is increasing evidence that GSRs could also be expected at exit wounds. Di Maio reports that especially cylindrical gunpowder particles can travel along the whole bullet path and can therefore be found at the exit area [14]. Große Perdekamp et al. showed in their experimental setting with gelatine blocks and pigskin that the highest amount of GSRs is found around the bullet entry and decreases along the shot channel, whereas it increases again at the bullet exit region. They proved the existence of GSRs along the whole bullet path. In contact shots, this phenomenon was explained with the formation of a temporary cavity that leads to an additional energy release with consequently pumping gunpowder particles to both directions (bullet entry and exit) after its collapse. Thus, burned and unburned flake-shaped particles are spotted around the exit region [18].

For this report, we present a case, where a similar uncommon pattern of unburned GSRs has been found in the hair around the exit wound after a fatal headshot of an 84-year-old male.

## Casuistic

**Fig. 1:**
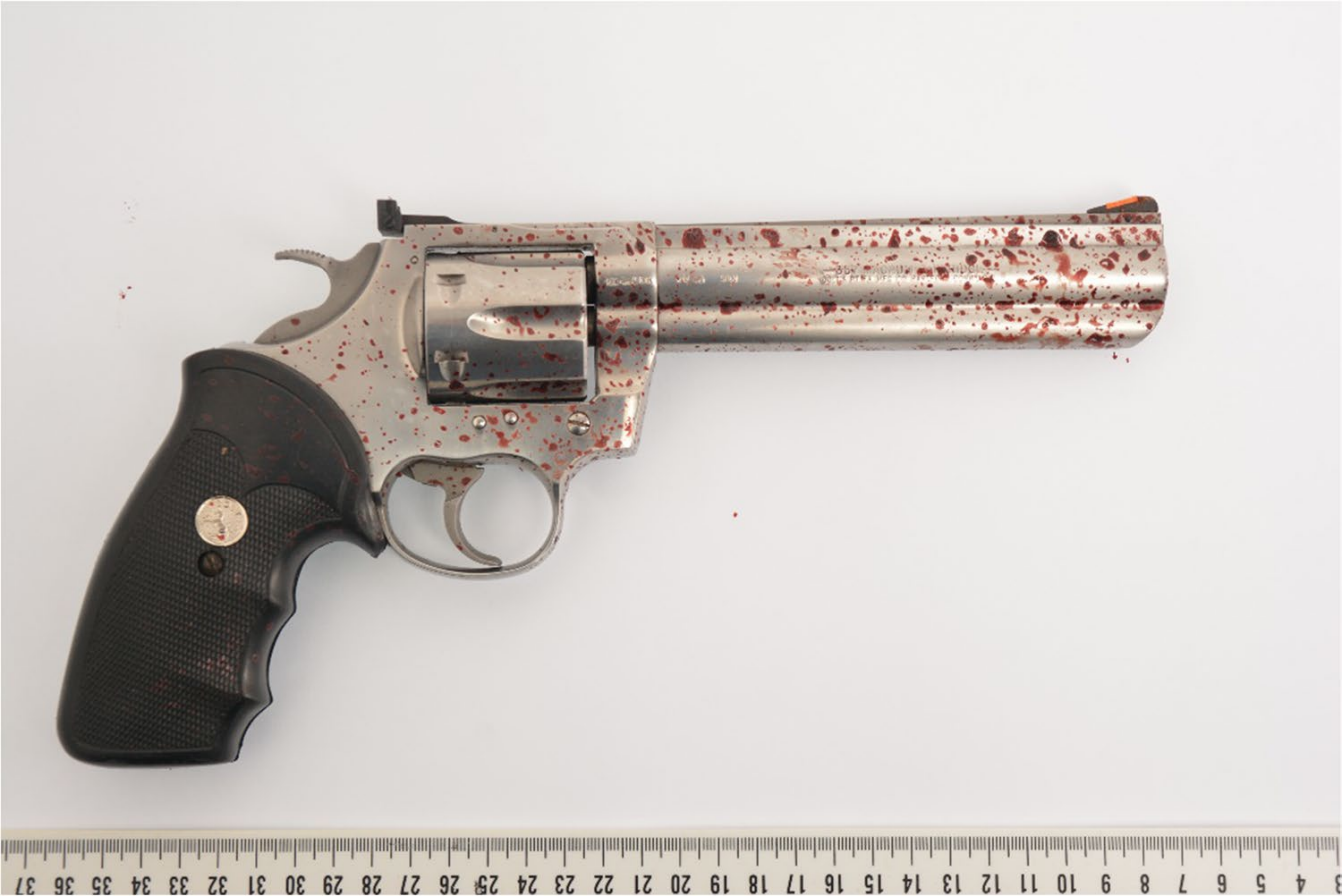
Weapon found at the location of incident

After she heard the noise of a gunshot, a woman found her 84-year-old husband sitting in a chair with a bleeding head wound in their cellar. The consulted emergency doctor confirmed his death. A revolver, model Colt (Fig. 1), type King Cobra and calibre 357 Magnum, which has been owned legally by the 84-year-old, has been found near the body. A cartridge case, calibre 38 Special named GECO from the manufacturer RUAG Ammotec GmbH, was located in the cylinder as a leftover of the fired cartridge. In detail, it was a flat nose semi-jacketed bullet with a bullet mass of 10.2 g.

**Fig. 2:**
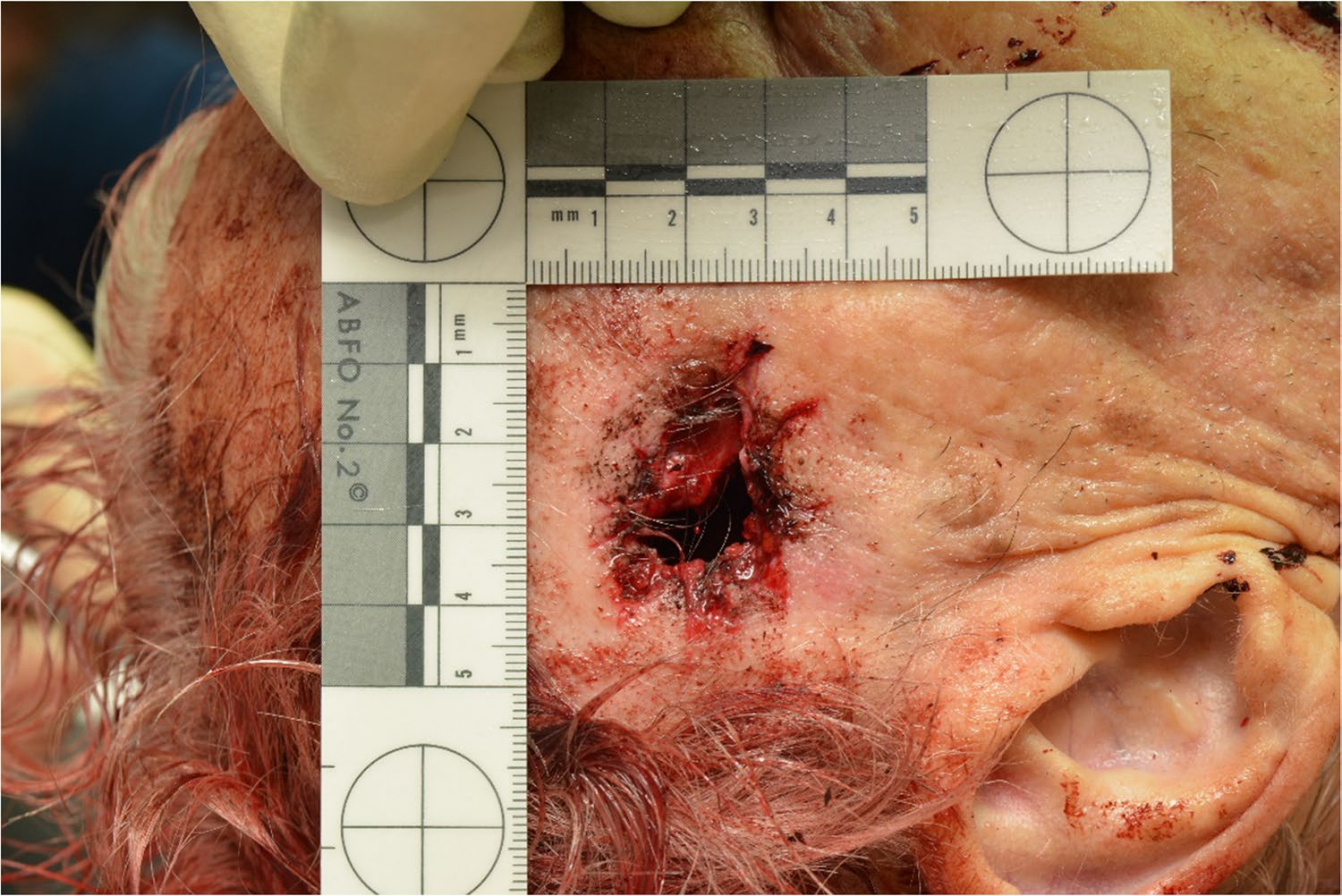
Gunshot entrance wound showing a lacerated wound with black soot visible within the wound and on the wound margins

**Fig. 3:**
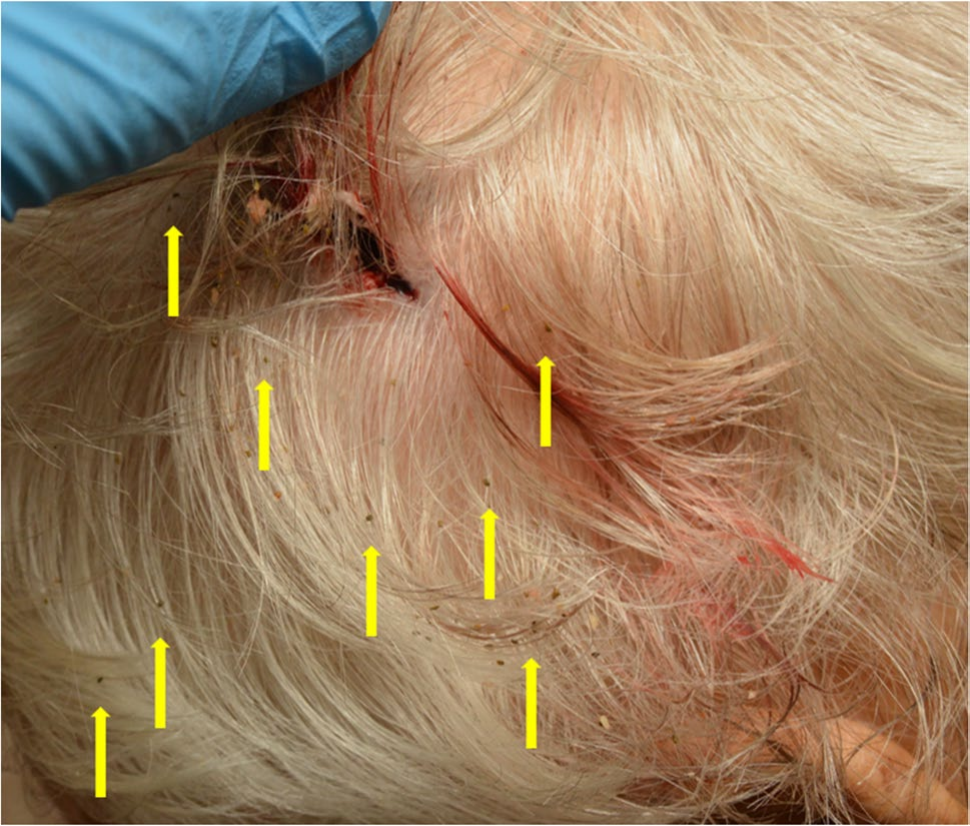
GSRs found around the exit wound (yellow arrows)

**Fig. 4:**
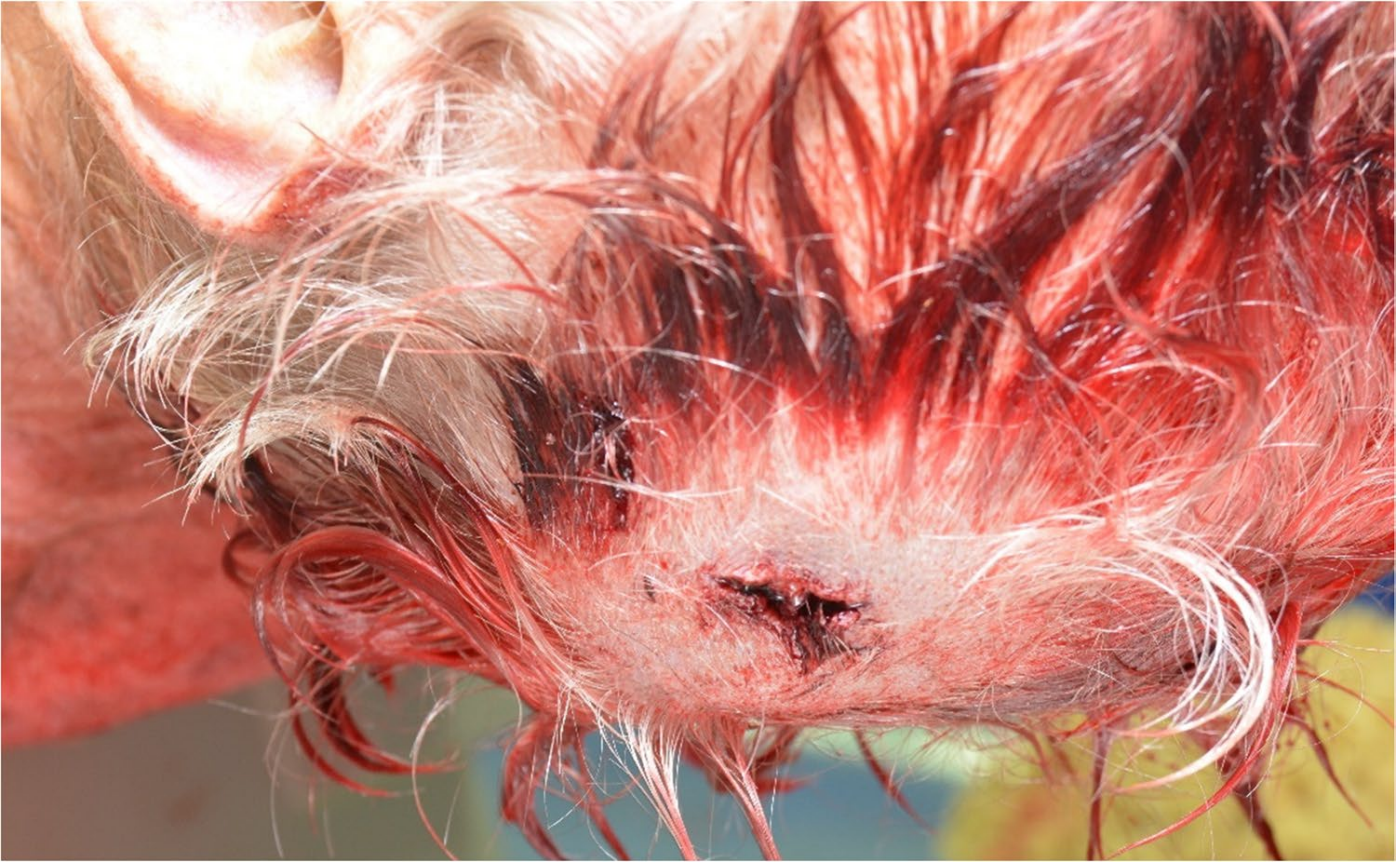
Impression of the exit wound during autopsy

**Fig. 5:**
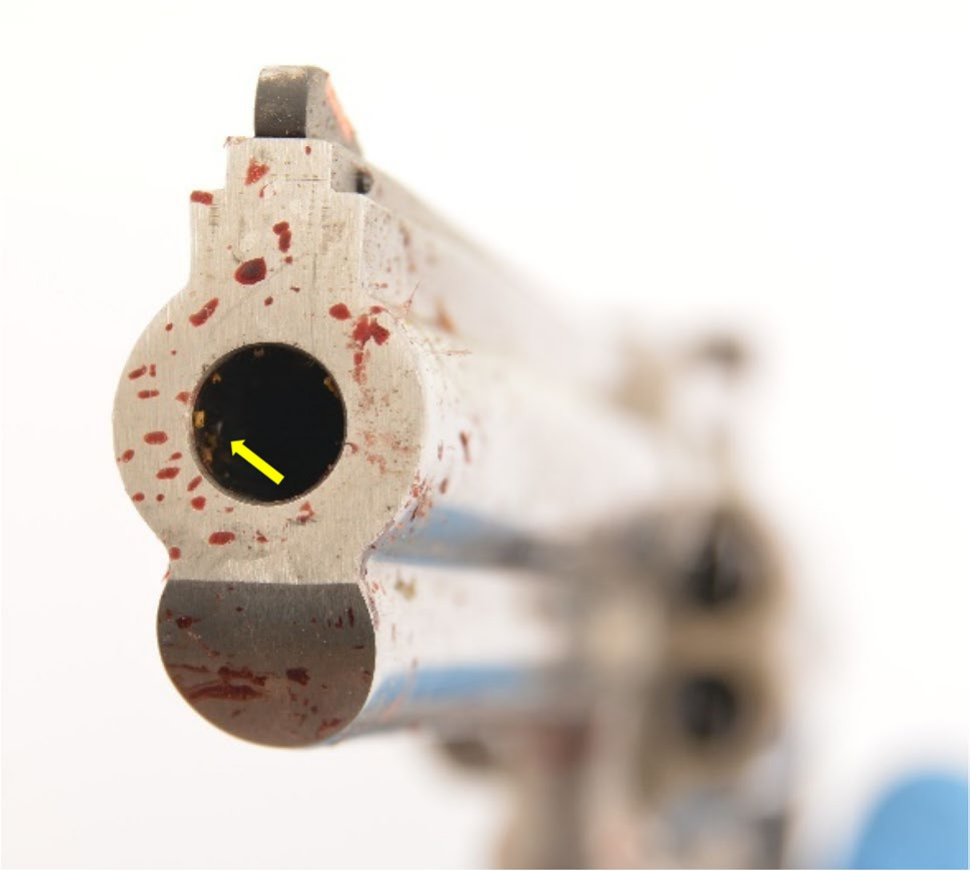
GSRs at the inside of the muzzle (yellow arrow)

The projectile’s trajectory passed the head entering the front right temple area and left the head slightly upwards at the high left occiput before it perforated an aluminium ladder and finally hit the wall. Autopsy findings correlated with an absolute close-range shot, with the presence of the typical blast injury, slight muzzle imprint mark and deposition of soot in the subcutaneous tissue (Fig. 2). Circular around the exit wound in the man’s hair, we found blood and tissue particles and interestingly also several flake-formed, partly cuboid or tube-like, greenish powder particles with a size less than 1 mm (Fig. 3). Noteworthy, these particles could hardly be observed during autopsy as most of the particles have probably been washed out during body transport (Fig. 4), but could be seen with a close look at the scene photos provided by the police. Additionally, these photos show similar particles adhered to the muzzle (Fig. 5). These findings moreover outline the importance of having a close look at incidence scene photos and to get these in good picture quality.

## Ballistic background

Powder burn-up in a weapon is highly dependent on the powder used and its ignition. In short-barrelled weapons, a fast-burning powder is commonly used so that the burning is as complete as possible when the bullet leaves the barrel. With very short-barrelled weapons, it must nevertheless be expected that part of the propellant charge leaves the barrel unburned or partly burned behind the bullet. The same can also happen with long-barrelled handguns if old or badly stored cartridges are fired, e.g. if the powder or primer has become humid, or if insufficient quality ammunition is used.

**Fig. 6:**
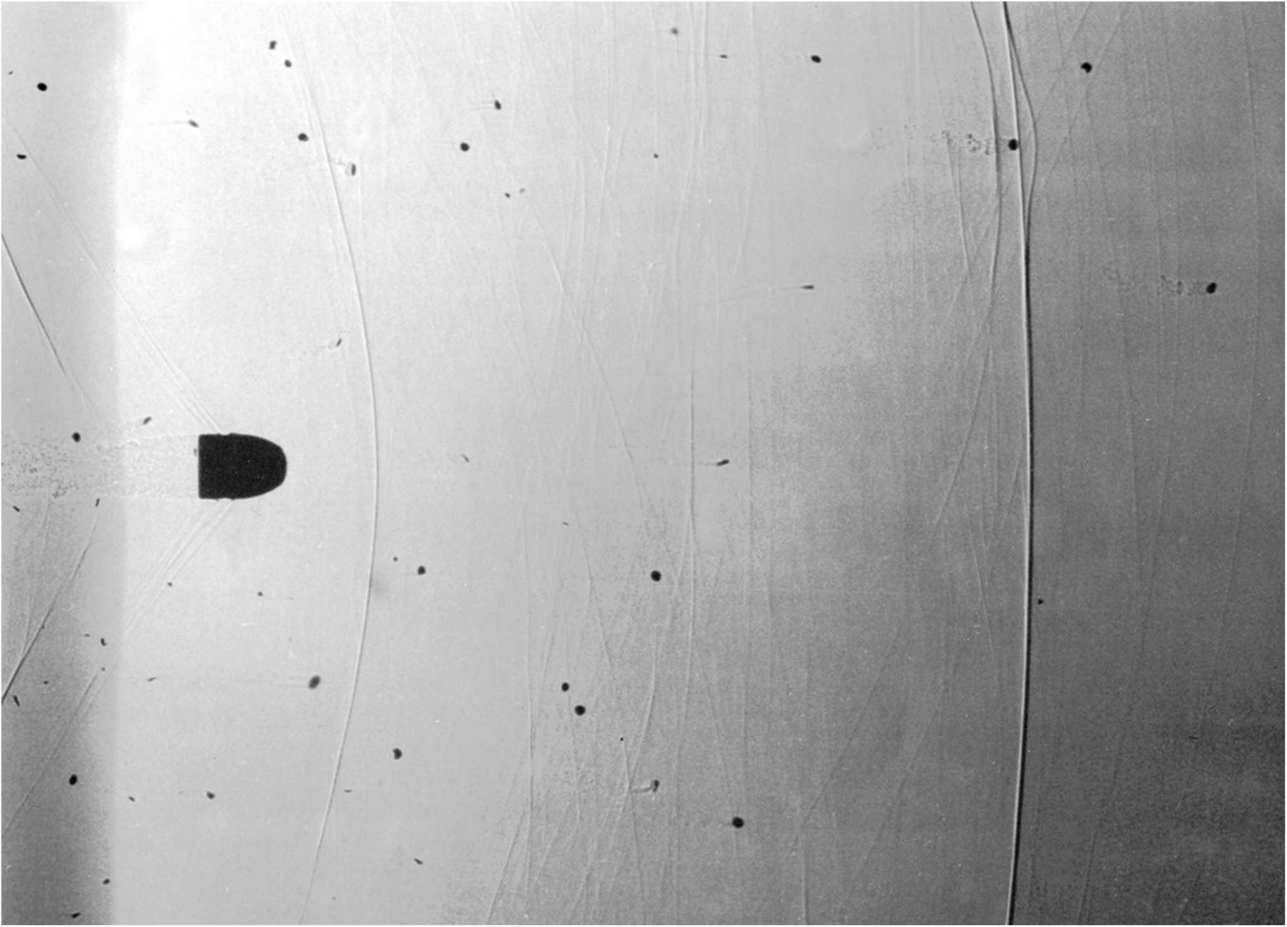
Some powder particles initially fly faster than the projectile before they are all finally overtaken by the projectile

If such a shot is fired with a free muzzle, these powder particles initially fly faster than the bullet and pass it until they are overtaken by the bullet after a few 10 cm (Fig. 6). In the case of a contact shot, they flow behind the bullet into the forming shot channel and thus enter the area of the temporary cavity, which still opens even if the bullet has already exited again in the case of a shot channel that is not too long. When the temporary cavity collapses, the powder particles are ejected again (together with powder soot and other particles sucked in at the same time), whereby this is possible through both existing openings (entry and exit hole).

## Discussion

**Fig. 7:**
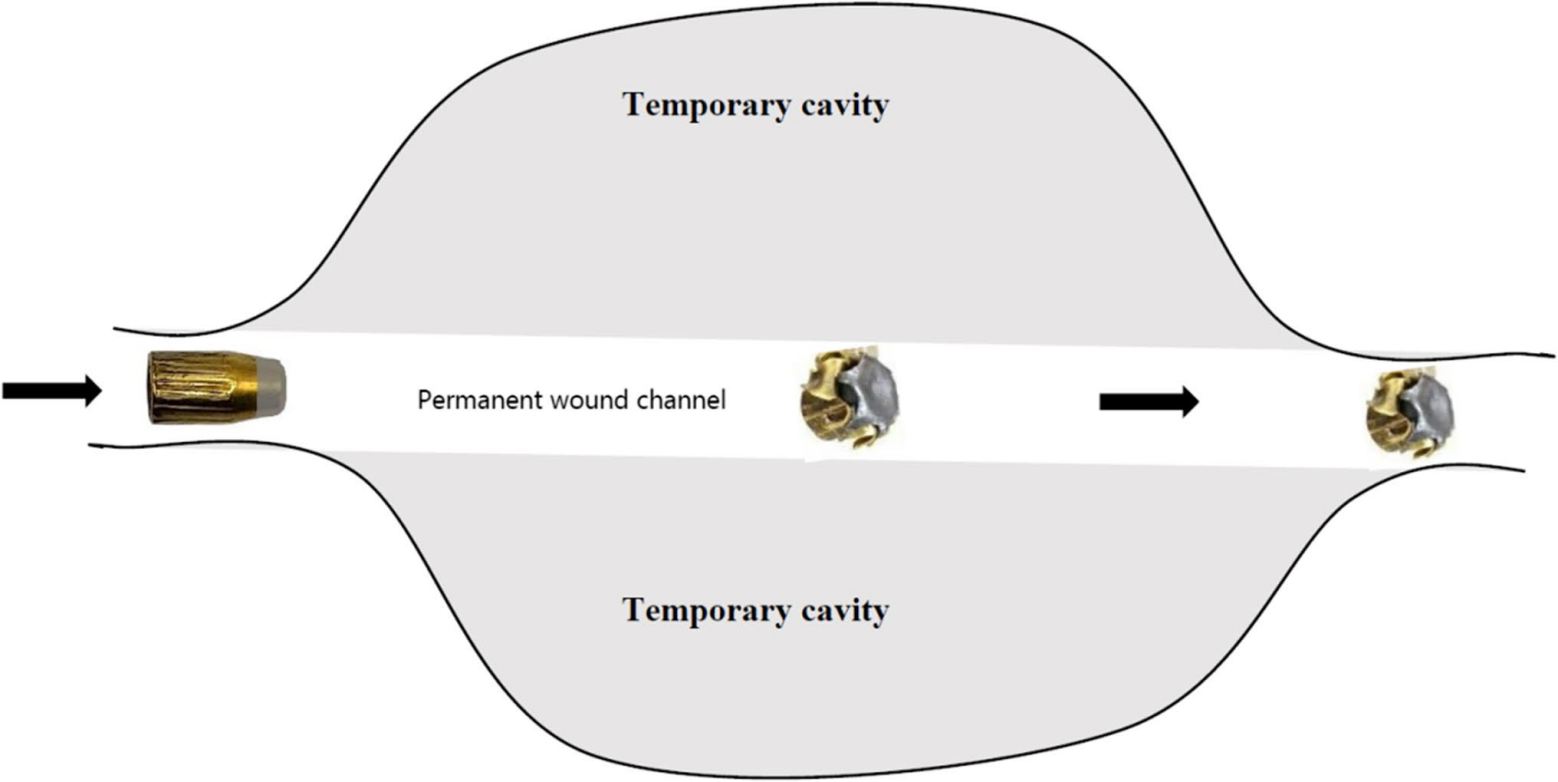
Entry of the bullet causing the formation of a temporary cavity located around the permanent wound channel (bullet illustrations were taken from [20, 21], modified and used for creation of this graph)

The differentiation of entrance and exit wounds by determining the presence or absence of GSRs is outdated, and ballistic principles are far more complex. By looking at the distribution of GSRs along the wound channel, it is possible in most cases to determine the direction in which the bullet has been travelling, as the concentration of GSRs usually decreases over the length of the bullet path. Additionally, the same is true for textile fibres. Due to the lower pressure behind the bullet, particles and fibres are sucked into the wound, whereas the vacuum created by the temporary cavity (Fig. 7) sucks them in even further and its collapse finally leads to their distribution to both openings, entry as well as exit wound. Therefore, it is not unusual to find textile fibres as well as GSRs at the exit wound [19]. This is also frequently observed in revolvers, as the gap between the cylinder and the barrel causes a pressure drop which delays the burning of the powder.

Moreover, it has already been shown that not only those particles are transported along the bullet path, but also skin particles have been found all the way along the wound channel [22]. Große Perdekamp et al. showed that even skin bacteria are transported from bullet entrance to the exit wound [23], and surprisingly, this has also been shown vice versa [24], which underlines the suction effect caused by the formation of a temporary cavity. They assume that this effect is influenced by the bullet properties, the local energy transfer to the tissue as well as the length of the bullet track [24]. In the mentioned studies, they show that — same as with GSRs — also skin particle and bacterial concentration decrease along the bullet path before those increase again at the exit area. Despite this evidence, the described circumstances and ballistic pattern of the reported case are still uncommon.

In the present case, a cartridge in 38 Special calibre was fired with a bullet from a Revolver Model Colt with a barrel length of 15 cm. This barrel length should actually result in an almost complete burn-up of gunpowder. This justifies the assumption that an older cartridge was used in this case. This is also indicated by the surprisingly large number of unburned and partially burned powder particles present at the exit wound of this injury. The powder particles that have been found around the exit wound showed flake-formed shape. In contrast to that, at the inside of the muzzle (Fig. 5), one can see that the powder particles appear tubular shaped or even like macaroni powder particles. These types of powder particles burn inside out and outside in. An incomplete burn-up of this kind of particles will lead to the formation of thin tubes, which can disintegrate to flake-formed particles like those seen at the exit wound. From the catalogues of RUAG (manufacturer of Geco ammunition) available to us, we can see that production of the 38 Special semi-jacketed flat nose cartridge was discontinued between 2007 and 2013. As the ammunition box has not been found at the crime scene, we do not have a lot number referring to the definite manufacturing date and we are therefore not able to conclude which kind of powder has been loaded. Nevertheless, after our investigations, we can conclude that the ammunition must have been purchased before 2013 and has therefore been stored for at least nine years. Another aspect that needs to be taken into consideration is the fact that a calibre 38 Special cartridge is shorter than the chambers in the cylinder of a 357 Magnum Revolver. This leads to a free flight of the bullet over a distance of a few millimetres and, together with the gap between the barrel and the cylinder, results in a pressure drop that influences the burning of the powder and explains the large number of unburned and partly burned powder grains [25]. Because of the possibility of detecting powder particles and smoke on the bullet exit under such conditions, special care must be taken when determining entry and exit gap and thus the direction of firing. Furthermore, a potential contamination of GSR from the entry region must be taken into consideration. In our case, the body has not been moved before taking the scene photos, nor has anything been changed at the scene. The body has been moved after completed crime scene investigation by the mortician and was subsequently packed in a fresh body bag. As GSRs have been probably washed out during body transport, analysis of powder particles at the Institute of Legal Medicine was not possible.

Gunpowder “tattooing” of the skin is definitely unique for bullet entry regions and not found at exit wounds. In summary, the findings within this report show that it is crucial to be familiar with ballistic aspects and to have a close look at scene photographs as wound pattern may have changed at the time of autopsy.
